# Editorial for *Brain Sciences* Special Issue: “Diagnosis of Neurogenetic Disorders: Contribution of Next-Generation Sequencing and Deep Phenotyping”

**DOI:** 10.3390/brainsci9030072

**Published:** 2019-03-26

**Authors:** Alisdair McNeill

**Affiliations:** Department of Neuroscience, University of Sheffield, 385a Glossop Road, Sheffield S10 2HQ, UK; a.mcneill@sheffield.ac.uk

In this Special Issue we bring together papers demonstrating the need for both detailed genomic and phenotypic studies to aid our scientific and clinical understanding of neurogenetic disorders. Genomic techniques such as genome and exome sequencing are vital tools for diagnosing rare neurogenetic disorders and identifying novel causal genes [1,2]. In this Special Issue, Gardner and colleagues [3] utilise genomic techniques to identify a series of individuals with brain malformations due to the recurrent *TUBA1A p.Arg2His* variant. This paper also describes the detailed phenotyping required to aid in the interpretation of novel genomic variants. Variants in *GBA1*, the gene causing Gaucher Disease (GD), are associated with an increased risk of Parkinson’s Disease (PD) [4,5]. Gatto and colleagues review this important area and describe how simple clinical observations helped begin the identification of this important risk factor for PD [6]. Genomic techniques are crucial for the diagnosis of neurogenetic disorders [7]. Garcia and Bustos [8] review the impact of such techniques for both the diagnosis of neurogenetic diseases and increasing our scientific understanding of these disorders. It is only through the co-development of both novel genomic and phenotypic assessments that we will be able to fully understand the pathogenesis of neurogenetic disorders and identify novel treatments.
